# Some Unusual Swellings of the Abdominal Parietes

**Published:** 1909-04-24

**Authors:** 


### SOME UNUSUAL SWELLINGS OF THE ABDOMINAL PARIETES.

Swellings of the abdominal parietes in the form of one of the varieties of hernia are, of course, of very common occurrence, but one occasionally comes across tumours and fluctuating swellings, quite unconnected with a hernia, whose diagnosis is not at all a simple matter. The first point which has to be made out is whether the tumour is in the parietes or is intra-abdominal and only causes a swelling by pushing forward the muscular wall in front of it; and the same difficulty presents itself in the case of a fluctuating swelling.

All such swellings should be examined first of all with the patient lying flat on his back and the abdominal muscles as much relaxed as possible. Many patients, especially nervous ones, persistently hold the abdominal muscles rigid; but it will generally be found that if the examination be conducted gently and without haste, the confidence of the patient may be gained and he will then relax the muscles. To ensure this it is a good plan to engage him in conversation during the examination, and so distract his attention from his abdominal wall, and it is also of some assistance in achieving this object to tell him to breathe in and out quietly. When the abdominal wall is relaxed, the mobility of the swelling can be estimated, *i.e.* whether it moves from side to side with the rectus abdominis or not.

If he is then told to raise his head from the bed, the muscles will at once contract, and a tumour, which is intimately connected with them, will become quite immovable. Or, again, if the tumour lies between the muscles and the superficial tissues, its outline usually becomes more distinct.

The patient should next be examined in the genu-pectoral position. In this way it can be ascertained whether the swelling moves with the abdominal wall in the antero-posterior plane.

Again, if the swelling fluctuates, the patient should be made to cough, and the swelling should be examined for the presence of an impulse. All fluctuating swellings of the abdominal wall, when examined in this way, will give an impulse which is communicated by the contraction of the muscles; but an expansile impulse will only be given by those swellings which are primarily intra-abdominal, and part of which has come to the surface either by thinning out the muscle wall in front of it or by eroding it.

Finally, the contour of the tumour should be carefully examined, as this may give an indication as to which muscular stratum it is connected with.

A rare form of solid tumour which occasionally occurs in the rectus abdominis is a diffuse fibroma. This starts as a localised swelling in one of the compartments of the muscle, and then tends to spread in each direction up and down. At each of the intersections of the muscle it is constricted, the appearance thus being presented of a chain of separate tumours, extending in advanced cases from the symphysis pubis to the costal arch, and it is sometimes known as a "desmoid" tumour for this reason. Excision of the mass is impracticable, since the rectus is completely replaced by tumour formation, and any attempt to remove it would result in excessive weakness of the parietes and a subsequent ventral hernia.

Fluctuating swellings in the neighbourhood of the umbilicus, other than a hernia, are not very rare, but are apt to be misleading, especially in adults. Occasionally the skin in this region becomes irritated or abraded, and septic infection of the subcutaneous tissues follows, leading to a peri-umbilical abscess. This causes a fluctuating swelling spreading from the navel in one or more directions. It exhibits all the phenomena of acute inflammation, *i.e.* it is tender, hot, and the skin covering it is suffused with a purplish-red colour. Incision and evacuation of the pus, followed by fomentations, will usually lead to a rapid subsidence of the symptoms. In infants a fluctuating peri-umbilical swelling is most often the result of tuberculous peritonitis; the disease starts in the mesenteric glands, which caseate, and an intra-peritoneal abscess is formed, which makes its way to the surface at the weakest spot, which is the umbilicus. Such swellings give a true expansile impulse on coughing. Unless they are opened early, they tend to burst spontaneously, and a chronic discharging sinus is left. Subsequent treatment consists in increasing the resistance of the child to the tubercle bacillus by vaccine therapy and improving its general health.

But while a tuberculous peritoneal focus, if it makes its way to the surface at all, usually does so at the umbilicus, it may rarely become superficial at some other spot; and then the diagnosis is a matter of greater difficulty. It may present in the epigastrium, where it may simulate a perigastric abscess, or in the neighbourhood of the cæcum, where it may resemble an appendix abscess. It is, of course, quite rare nowadays, when appendicitis is operated on as soon as diagnosed, to see a case in which an appendix abscess has been allowed to make its own way to the surface. But even now it is not unknown.

The introduction of antitoxin, especially diphtheria antitoxin, into the tissues of the abdominal wall is sometimes responsible for swellings in this neighbourhood. Some of these are due to the fact that the antitoxin is not absorbed but becomes encysted *in situ*; but more frequently the swelling results from injection with a needle which is not absolutely sterile, and a septic infection of greater or less severity is set up. When this occurs in the right iliac fossa the case may present superficial points of similarity to one of appendicitis, but the extraction of an accurate history should clear up the diagnosis. Quite recently the writer saw a child, aged six, with a diffuse tender swelling over the appendix, which the mother said had existed for four or five days. He looked ill, had vomited twice, his temperature was 101.5°, and his pulse 110. It transpired that the child had been discharged from a fever hospital a fortnight previously, where he had been treated for diphtheria with antitoxin. The swelling was incised, and proved to be an abscess of a not very acute type. It is presumable that in this case the antitoxin was not absorbed after injection, but became encysted, and that auto-infection occurred later. Immediately after operation the symptoms all subsided, and recovery was rapid and uneventful.

